# Comparative Effects of SGLT2 Inhibitors and GLP-1 Receptor Agonists on Composite Surrogate Markers of Insulin Resistance: A Real-World Study Using METS-IR and SPISE

**DOI:** 10.3390/jcm15124403

**Published:** 2026-06-06

**Authors:** Dimitra Voziki, Ioannis Stergiou, Ioanna Zografou, Maria Mavridou, Lefteris Teperikidis, Michael Doumas, Evangelos N. Liberopoulos, Kalliopi Kotsa, Matilda Florentin, Theocharis Koufakis

**Affiliations:** 1 Department of Internal Medicine and Diabetes Clinic, “G. Gennimatas” General Hospital, 54635 Thessaloniki, Greece; 2 Second Propaedeutic Department of Internal Medicine, Medical School, Hippokration General Hospital, Aristotle University of Thessaloniki, 54642 Thessaloniki, Greece; 3 Clinical Research Unit, Special Unit for Biomedical Research and Education (SUBRE), School of Medicine, Aristotle University of Thessaloniki, 54124 Thessaloniki, Greece; 4 First Department of Propaedeutic Internal Medicine, Medical School, National and Kapodistrian University of Athens, Laiko General Hospital, 11527 Athens, Greece; 5 Division of Endocrinology and Metabolism and Diabetes Center, First Department of Internal Medicine, Medical School, Aristotle University of Thessaloniki, AHEPA University Hospital, 54636 Thessaloniki, Greece; 6 Faculty of Medicine, School of Health Sciences, University of Ioannina, 45110 Ioannina, Greece

**Keywords:** type 2 diabetes, METS-IR, SPISE, SGLT2 inhibitors, GLP-1 receptor agonists, cardiometabolic risk, surrogate markers

## Abstract

**Objective:** Insulin resistance is a key pathophysiological driver linking obesity and type 2 diabetes (T2D) with cardiovascular risk. Composite surrogate indices derived from routine clinical parameters, such as the Metabolic Score for Insulin Resistance (METS-IR) and the Single Point Insulin Sensitivity Estimator (SPISE), may provide a practical means of capturing multidimensional metabolic changes. Given that comparative data are limited, we aimed to evaluate the effects of sodium–glucose cotransporter-2 inhibitors (SGLT2i) and glucagon-like peptide-1 receptor agonists (GLP-1RA) on these indices in individuals with T2D and overweight or obesity. **Methods:** In this retrospective observational study, 100 individuals with T2D treated with either GLP-1RA (*n* = 54) or SGLT2i (*n* = 46) were evaluated over 6 months. Strict inclusion criteria ensured treatment stability without initiation or modification of concomitant pharmacotherapy. Changes in METS-IR and SPISE were assessed alongside body mass index (BMI) and glycated hemoglobin (HbA1c). Multivariable regression and exploratory analyses, including stratification by BMI and correlation analyses, were performed. **Results:** Both treatment groups demonstrated significant improvements in METS-IR (GLP-1RA: −3.9 ± 5.9; SGLT2i: −2.5 ± 2.6; both *p* < 0.001) and SPISE (GLP-1RA: +0.46 ± 0.52; SGLT2i: +0.44 ± 0.61; both *p* < 0.001), with no significant between-group differences. In the GLP-1RA group, changes in METS-IR correlated with changes in BMI (r = 0.48, *p* < 0.001) and HbA1c (r = 0.29, *p* = 0.030), whereas no significant correlations were observed in the SGLT2i group. Stratified analyses indicated greater reductions in METS-IR among individuals with BMI ≥30 kg/m^2^ treated with GLP-1RA. **Conclusions:** Both SGLT2i and GLP-1RA improve composite surrogate markers of insulin resistance, with distinct associations with weight and glycemic changes. METS-IR and SPISE may serve as practical tools for monitoring multidimensional metabolic responses in clinical practice.

## 1. Introduction

Type 2 diabetes (T2D) is a major contributor to cardiovascular morbidity and mortality worldwide [1]. Beyond hyperglycemia, insulin resistance plays a central role in the pathophysiology of T2D, contributing to dyslipidemia, endothelial dysfunction, inflammation, and alterations in adipose tissue distribution [2]. Together, these processes create an atherogenic milieu that is not adequately reflected by glycemic indices alone, highlighting the need for broader assessment of cardiometabolic risk in this population [3].

In recent years, the therapeutic landscape of T2D has undergone a paradigm shift with the introduction of sodium–glucose cotransporter-2 inhibitors (SGLT2i) and glucagon-like peptide-1 receptor agonists (GLP-1RA). These agents have consistently demonstrated cardiovascular and renal benefits in large outcome trials, leading to their prioritization in contemporary guidelines independently of baseline glycemic control [4]. Importantly, their mechanisms of action extend beyond glucose lowering, encompassing effects on body weight, hemodynamics, lipid metabolism, and insulin sensitivity, which together drive their clinical benefits, although the relative contribution of these pathways may differ between drug classes [5].

In this context, there is increasing interest in the use of surrogate markers that capture the complex interplay between insulin resistance, adiposity, and cardiovascular risk. Direct assessment of insulin resistance using gold-standard techniques such as the hyperinsulinemic–euglycemic clamp is not feasible in routine clinical practice, while even simpler indices requiring insulin measurements are not universally available [6]. Therefore, composite indices derived from routinely measured clinical and biochemical variables have emerged as practical tools for estimating insulin resistance and related metabolic burden in real-world settings [7]. Among these, the Metabolic Score for Insulin Resistance (METS-IR) and the Single Point Insulin Sensitivity Estimator (SPISE) have gained attention as validated, non-insulin-based indices that integrate anthropometric and lipid parameters with glycemic measures [8]. These indices have been shown to associate with cardiometabolic risk across diverse populations, making them attractive candidates for use in both clinical and research settings [9]. Furthermore, both indices have demonstrated significant correlations with established insulin-based measures of insulin resistance and insulin sensitivity, including Homeostatic Model Assessment for Insulin Resistance (HOMA-IR) and clamp-derived indices, supporting their validity as practical surrogate markers when insulin measurements are unavailable [10].

The present study aimed to comparatively evaluate the effects of treatment with SGLT2i and GLP-1RA on METS-IR and SPISE over a 6-month period in individuals with T2D. Additionally, we sought to examine the relationship between these indices and changes in body weight and glycemic control, and to assess whether baseline obesity status modifies these associations.

## 2. Methods

### 2.1. Study Design and Population

This retrospective cohort study utilized routinely collected clinical data from adults with T2D attending two tertiary diabetes clinics in Thessaloniki, Greece (Hippokration General Hospital and G. Gennimatas General Hospital). Eligible individuals were identified from medical records of patients who initiated treatment with either a GLP-1RA or an SGLT2i between March 2024 and June 2024 and completed 6 months of follow-up. Retrospective review of medical records and data extraction were performed between December 2024 and February 2025. The analysis was conducted in the same study population as a previously published investigation examining the effects of SGLT2i and GLP-1RA on the triglyceride-to-HDL cholesterol ratio and the triglyceride–glucose (TyG) index [11], but addresses a distinct research question focusing on multidimensional composite markers integrating glycemic, lipid, and anthropometric parameters, namely METS-IR and SPISE.

Adults with established T2D receiving follow-up at the participating clinics were screened for eligibility. Inclusion required initiation of either a GLP-1RA or an SGLT2i and availability of complete clinical and biochemical assessments at treatment initiation and approximately 6 months later. All management decisions, including lifestyle counseling and follow-up scheduling, reflected routine clinical practice [12].

To reduce potential confounding, patients were excluded if changes occurred in medications known to influence the metabolic variables incorporated into the investigated indices. Exclusion criteria included initiation of additional glucose-lowering, lipid-lowering, or antihypertensive therapies, modification of existing antidiabetic treatment regimens, and inadequate treatment adherence or follow-up. These restrictions were implemented to maximize attribution of observed changes to the study medications.

### 2.2. Data Collection and Study Variables

Information was extracted from medical records at treatment initiation and after 6 months of follow-up. Recorded variables included age, sex, body weight, height, BMI, fasting plasma glucose, HbA1c, total cholesterol, triglycerides, LDL cholesterol, and HDL cholesterol.

METS-IR was calculated as previously described by Bello-Chavolla et al. [13], incorporating FPG, triglycerides, HDL cholesterol, and BMI into a composite index reflecting insulin resistance and cardiometabolic risk, with higher values indicating greater insulin resistance (i.e., a less favorable metabolic profile). SPISE was calculated according to the method proposed by Paulmichl et al. [14], based on HDL cholesterol, triglycerides, and BMI, with higher values indicating greater insulin sensitivity (i.e., a more favorable metabolic profile). Indices were calculated at baseline and at 6 months. A decrease in METS-IR and an increase in SPISE were interpreted as favorable metabolic changes [15].

### 2.3. Statistical Analysis

Data distribution was evaluated using the Shapiro–Wilk test. Continuous variables are reported as mean ± standard deviation, whereas categorical variables are presented as absolute frequencies and percentages. Baseline differences between treatment groups were assessed using independent-samples *t*-tests or Mann–Whitney U tests, depending on data distribution. Comparisons of categorical variables were performed with the χ^2^ test. Longitudinal changes within each treatment group were evaluated by paired-samples *t*-tests or Wilcoxon signed-rank tests, as appropriate. For all continuous variables, change (Δ) was defined as the value at 6 months minus the corresponding baseline value. Differences in Δ values between the GLP-1RA and SGLT2i groups were examined using independent-samples *t*-tests or Mann–Whitney U tests according to distributional characteristics. A responder analysis was additionally performed. Improvement was defined as a decrease in METS-IR, an increase in SPISE, or improvement in both indices from baseline to 6 months. Responder proportions were compared between treatment groups using the χ^2^ test.

To account for baseline differences and potential confounding inherent to the retrospective design, multivariable linear regression models were constructed with 6-month METS-IR and SPISE as dependent variables. Independent variables included treatment group (GLP-1RA vs. SGLT2i), baseline value of the respective index, age, baseline BMI, and baseline HbA1c. Regression coefficients (β), 95% confidence intervals (CI), and *p*-values were reported. A sensitivity analysis was performed by additionally including change in BMI (ΔBMI) and change in HbA1c (ΔHbA1c) in the regression models to explore whether treatment effects were independent of weight loss and glycemic improvement.

Exploratory analyses included stratification according to baseline BMI (<30 vs. ≥30 kg/m^2^), with between-group comparisons of Δ values conducted within each stratum. These analyses were considered hypothesis-generating. In addition, Pearson correlation coefficients were calculated to assess the associations between ΔMETS-IR and ΔBMI, as well as ΔMETS-IR and ΔHbA1c, separately within each treatment group. Differences in correlation coefficients between groups were evaluated using Fisher’s z-transformation. All analyses were performed using Python (version 3.11) with the pandas, NumPy, and SciPy libraries. All statistical tests were two-sided, and a *p*-value < 0.05 was considered statistically significant. Formal sample size calculations were not performed because the study was designed as a retrospective exploratory analysis. Post hoc power analyses were also not conducted, as they are generally considered to provide limited additional interpretative value in observational research [16].

### 2.4. Ethical Considerations

All study procedures were performed in accordance with the ethical principles of the Declaration of Helsinki. The study protocol was reviewed and approved by the Bioethics Committee of the Medical School of Aristotle University of Thessaloniki (approval no. 24; 15 November 2024). Written informed consent had been obtained from all participants, allowing the use of anonymized clinical and laboratory data for scientific research.

## 3. Results

### 3.1. Study Population, Baseline Characteristics and Metabolic Changes

**Figure 1:**
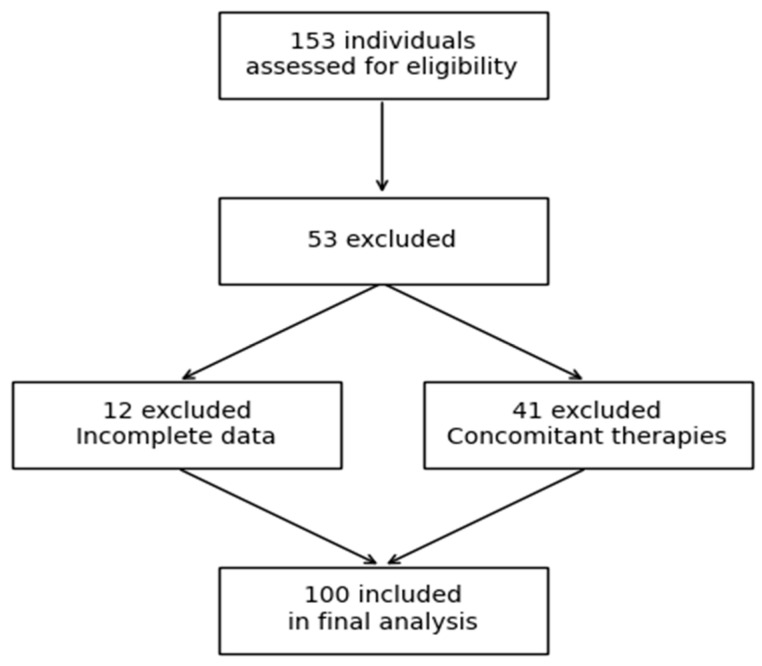
Flow diagram of participant selection and study inclusion.

A total of 153 potentially eligible individuals were screened, reflecting the number of patients available at the participating hospitals during the study period. Of these, 12 were excluded because of incomplete baseline and/or follow-up data, while 41 were excluded because of initiation of concomitant pharmacologic therapies during follow-up that could affect the metabolic indices under investigation. The final study population consisted of 100 individuals fulfilling all eligibility criteria, comprising 54 treated with GLP-1RA (dulaglutide or semaglutide) and 46 with SGLT2i (empagliflozin or dapagliflozin). Participant selection is summarized in Figure 1. No treatment discontinuations occurred during the observation period, possibly reflecting follow-up in specialized diabetes clinics with experience in the initiation and monitoring of contemporary glucose-lowering therapies.

Individuals receiving GLP-1RA were younger, whereas sex distribution was similar. As shown in Table 1, compared with the SGLT2i group, participants treated with GLP-1RA exhibited greater adiposity and higher triglyceride concentrations despite broadly comparable glycemic parameters.

Detailed analyses of conventional anthropometric and glycemic outcomes from this cohort have been published elsewhere [10]. Therefore, only a brief summary is provided here to contextualize the findings relating to METS-IR and SPISE. In summary, both treatment groups demonstrated reductions in body weight and FPG, with statistically significant decreases observed for FPG in both the SGLT2i (*p* = 0.022) and GLP-1RA groups (***p*** < 0.001). Improvements in HbA1c were more modest and did not reach statistical significance in the original analysis, highlighting the heterogeneity of glycemic response in this real-world cohort.

Baseline METS-IR values were higher and SPISE values were lower in the GLP-1RA group compared with the SGLT2i group (both *p* < 0.001; Table 2). Over the 6-month follow-up, METS-IR decreased significantly in both the GLP-1RA (Δ −3.9 ± 5.9, *p* < 0.001) and SGLT2i groups (Δ −2.5 ± 2.6, *p* < 0.001). Similarly, SPISE increased in both groups (GLP-1RA: +0.46 ± 0.52, *p* < 0.001; SGLT2i: +0.44 ± 0.61, *p* < 0.001). In unadjusted between-group comparisons of Δ values, the magnitude of change did not differ significantly between treatment groups for either METS-IR (*p* = 0.115) or SPISE (*p* = 0.870).

In responder analyses, METS-IR improved in 44 of 54 individuals (81.5%) treated with GLP-1RA and in 33 of 46 individuals (71.7%) treated with SGLT2i. Similarly, SPISE improved in 45 of 54 individuals (83.3%) and 33 of 46 individuals (71.7%), respectively. Improvement in both indices was observed in 43 of 54 individuals (79.6%) in the GLP-1RA group and 29 of 46 individuals (63.0%) in the SGLT2i group.

### 3.2. Multivariable and Stratified Analyses

In multivariable linear regression analyses adjusting for baseline differences in metabolic profile and adiposity, including baseline index values, age, BMI, and HbA1c, treatment group was not independently associated with 6-month METS-IR (β = −0.45, 95% CI −2.13 to 1.22, *p* = 0.594) or SPISE (β = 0.15, 95% CI −0.10 to 0.40, *p* = 0.242). These findings remained materially unchanged after additional adjustment for ΔBMI and ΔHbA1c, suggesting that the observed improvements in composite indices were not independently driven by treatment class.

When stratified by baseline BMI, greater reductions in METS-IR were observed among individuals with BMI ≥ 30 kg/m^2^ treated with GLP-1RA (Δ −4.9 ± 6.1) compared with those with BMI < 30 kg/m^2^ (Δ −2.1 ± 4.0; *p* = 0.048). No significant difference between BMI strata was observed in the SGLT2i group (*p* = 0.334). Between-group comparisons within BMI strata did not reach statistical significance for METS-IR or SPISE.

### 3.3. Correlation Analyses of Changes in METS-IR and SPISE

In the GLP-1RA group, ΔMETS-IR was moderately correlated with ΔBMI (r = 0.48, *p* < 0.001) and weakly correlated with ΔHbA1c (r = 0.29, *p* = 0.030). In contrast, in the SGLT2i group, no significant correlations were observed between ΔMETS-IR and ΔBMI (r = 0.21, *p* = 0.180) or ΔHbA1c (r = 0.12, *p* = 0.410). Between-group comparison demonstrated a statistically significant difference in the strength of association between ΔMETS-IR and ΔBMI (*p* = 0.041), but not for ΔHbA1c (*p* = 0.278). Correlation analyses for SPISE did not demonstrate significant associations with changes in BMI or HbA1c in either treatment group (Table 3).

## 4. Discussion

In the present study, we comparatively evaluated the effects of SGLT2i and GLP-1RA on non–insulin-based surrogate markers of insulin resistance and cardiometabolic risk, specifically METS-IR and SPISE, over a 6-month period in individuals with T2D. We observed that both treatment strategies were associated with improvements in these indices, with distinct patterns of effect between the two drug classes. Importantly, changes in METS-IR were significantly associated with changes in both body weight and glycemic control, supporting its role as an integrative marker of metabolic improvement. To our knowledge, this is the first study to directly compare the effects of these two contemporary drug classes on METS-IR and SPISE, thereby extending the assessment of treatment response beyond traditional surrogate indices.

The potential clinical relevance of these findings extends beyond glycemic control. Insulin resistance and the compensatory hyperinsulinemia that frequently accompanies it are increasingly recognized as important contributors to cardiovascular disease, even before the onset of overt diabetes [17]. Chronic insulin resistance is associated with endothelial dysfunction, oxidative stress, low-grade inflammation, adverse adipose tissue remodeling, dyslipidemia, and hypertension, all of which promote atherosclerosis and cardiovascular risk [18]. Furthermore, compensatory hyperinsulinemia may exert direct vascular and hemodynamic effects, including enhanced sympathetic nervous system activity, sodium retention, and vascular smooth muscle proliferation [19]. In this context, improvements in surrogate markers of insulin resistance such as METS-IR and SPISE may reflect broader cardiometabolic benefits extending beyond glucose lowering. Although the present study did not assess cardiovascular outcomes, the observed improvements in these indices provide additional mechanistic support for the favorable cardiometabolic effects of SGLT2i and GLP-1RA reported in large cardiovascular outcome trials.

Our findings are consistent with accumulating evidence indicating that modern glucose-lowering therapies exert beneficial effects on insulin resistance–related pathways. Previous studies with GLP-1RA, including liraglutide, have demonstrated improvements in body weight, lipid metabolism, and surrogate markers of insulin resistance, effects that are thought to contribute to their anti-atherosclerotic properties [15]. A recent study reported that liraglutide treatment in individuals with metabolic dysfunction–associated liver disease was associated with significant improvements in triglyceride-based indices of insulin resistance, including METS-IR, despite more modest changes in conventional markers [20]. These findings support the concept that composite indices may be particularly sensitive in capturing multidimensional metabolic improvements induced by GLP-1RA. Similarly, SGLT2i have been shown to improve surrogate markers such as the TyG index, although their effects on lipid-related parameters appear more heterogeneous [21].

Notably, although GLP-1RA is generally regarded as a more potent glucose-lowering agent, the comparable reductions in HbA1c observed in the present study likely reflect the influence of real-world treatment allocation and baseline patient characteristics rather than true equivalence in pharmacological efficacy. Individuals receiving GLP-1RA had higher baseline BMI and a less favorable metabolic profile, suggesting a greater degree of underlying insulin resistance, which may attenuate the relative glycemic response [22]. In addition, the relatively short follow-up period and the potential for incomplete dose titration in routine clinical practice may have further reduced detectable between-group differences. Conversely, the insulin-independent mechanism of action of SGLT2i may allow for consistent glycemic improvement across a broader spectrum of metabolic phenotypes [23]. Taken together, these considerations help explain the similar magnitude of HbA1c reduction despite differences in expected pharmacological potency and reinforce the importance of contextualizing treatment effects within real-world populations. A similar interplay of baseline differences and treatment dynamics may also underlie the comparable improvements observed in METS-IR and SPISE between groups.

From a clinical perspective, the use of simple, non–insulin-based indices such as METS-IR and SPISE may provide a pragmatic approach for monitoring the broader metabolic effects of contemporary glucose-lowering therapies. These indices rely on routinely available clinical variables and can therefore be readily incorporated into everyday practice without additional burden [24]. Their potential utility is further supported by previous validation studies demonstrating significant correlations with established insulin-based measures, including HOMA-IR and clamp-derived indices [8,10]. Nevertheless, the clinical interpretation of longitudinal changes in METS-IR and SPISE remains challenging because thresholds defining clinically meaningful improvement have not yet been established. Unlike isolated measures such as HbA1c or FPG, they capture multiple dimensions of cardiometabolic risk, including dyslipidemia and adiposity-related dysfunction. In this regard, our findings suggest that such indices may serve as complementary tools to traditional metrics, potentially enabling a more comprehensive evaluation of treatment response and supporting a more individualized approach to therapeutic decision-making [25].

An additional observation of interest is the apparent influence of baseline adiposity status on treatment response, as suggested by our exploratory analyses. When participants were stratified according to baseline BMI, differences in the magnitude of improvement in METS-IR were observed, indicating that the metabolic response to these therapies may vary across the spectrum of adiposity. This observation is biologically plausible, as increased adiposity—particularly when associated with central fat accumulation—is characterized by alterations in adipose tissue function, including chronic low-grade inflammation, dysregulated adipokine secretion, and ectopic lipid deposition, all of which contribute to insulin resistance [26]. GLP-1RA has been shown to influence several of these pathways, including reductions in visceral adipose tissue, improvements in inflammatory signaling, and modulation of energy balance, which may translate into more pronounced improvements in composite metabolic indices in individuals with higher levels of adiposity [27]. In contrast, the metabolic effects of SGLT2i may be mediated to a greater extent through mechanisms such as glycosuria-induced energy loss, hemodynamic changes, and shifts in substrate utilization [28]. These differences may partly explain the observed heterogeneity in treatment response across adiposity strata.

Furthermore, the finding that changes in METS-IR were more strongly associated with changes in BMI in the GLP-1RA group supports the hypothesis that weight reduction plays a central role in mediating improvements in insulin resistance–related pathways with this class of agents [29]. In contrast, the weaker associations observed in the SGLT2i group suggest that additional mechanisms, potentially independent of weight change, may contribute to metabolic improvements [30]. Collectively, these observations highlight the heterogeneity of insulin resistance and underscore the importance of considering underlying pathophysiological context when interpreting changes in surrogate markers. Nevertheless, given the exploratory nature of these analyses and the limited sample size, these findings should be interpreted with caution and require confirmation in larger, prospective studies.

The present study has several strengths, including the direct head-to-head comparison of two widely used drug classes, the use of composite indices that integrate multiple metabolic domains, and the application of strict inclusion criteria ensuring treatment stability throughout the study period. Notably, no additional pharmacological therapies were initiated and no treatment modifications were made during follow-up, allowing for a more isolated assessment of drug effects and minimizing potential confounding. However, important limitations should be acknowledged. The retrospective design introduces the possibility of residual confounding and selection bias, while the relatively modest sample size may limit statistical power, particularly for subgroup analyses. The observed baseline differences between groups, particularly in BMI and composite metabolic indices, likely reflect real-world treatment allocation and may have influenced the magnitude of observed changes despite multivariable adjustment and sensitivity analyses accounting for major baseline metabolic differences. In addition, data on lifestyle factors such as diet and physical activity were not systematically captured, although standardized recommendations were provided. Fasting insulin measurements were not available, precluding direct comparison of METS-IR and SPISE changes with insulin-based indices such as HOMA-IR. This was consistent with the study objective of evaluating markers derived from routinely available clinical variables and therefore reflecting real-world clinical practice rather than a research setting. Although both indices have been validated against established measures of insulin sensitivity, the absence of universally accepted thresholds for clinically meaningful longitudinal changes limits interpretation of the absolute magnitude of the observed improvements. Finally, the reliance on surrogate markers precludes direct conclusions regarding long-term cardiovascular outcomes. Accordingly, the findings should be interpreted primarily as real-world associations rather than evidence of comparative treatment efficacy.

## 5. Conclusions

In conclusion, both SGLT2i and GLP-1RA were associated with significant improvements in composite surrogate markers of insulin resistance, with distinct patterns of association with body weight and glycemic changes. These findings support the potential utility of METS-IR and SPISE as practical tools for capturing multidimensional metabolic responses in individuals with T2D. Future studies should aim to validate these findings in larger, prospective cohorts and to investigate whether changes in METS-IR and SPISE are associated with clinically meaningful endpoints, including cardiovascular events. In addition, mechanistic studies are warranted to further elucidate the differential pathways through which SGLT2i and GLP-1RA influence insulin resistance and adiposity-related processes. Ultimately, the integration of such surrogate markers into clinical trials and real-world practice may contribute to a more refined, precision-based approach to the management of T2D.

## Figures and Tables

**Table 1:** Baseline demographic, anthropometric and laboratory characteristics of the study population.

Parameter	SGLT2i (*n* = 46)	GLP-1RA (*n* = 54)	*p*-Value
Age (years)	66 ± 9.87	60.65 ± 10.62	0.011
Body weight (kg)	82.2 ± 17.9	98.1 ± 23.3	<0.001
BMI (kg/m^2^)	29.5 ± 6.1	34.4 ± 7.8	<0.001
FPG (mg/dL)	136 ± 37.4	145 ± 49.1	0.512
HbA1c (%)	7.1 ± 1.0	7.5 ± 0.8	0.362
TG (mg/dL)	151 ± 89.6	189 ± 62.1	<0.001
HDL-C (mg/dL)	46.1 ± 11.2	45.1 ± 11.2	0.241

Values are presented as mean ± Standard Deviation. Abbreviations: BMI: body mass index; FPG: fasting plasma glucose; HbA1c: glycated hemoglobin; TG: triglycerides; HDL-C: high-density lipoprotein cholesterol; GLP-1RA: glucagon-like peptide-1 receptor agonists; SGLT2i: sodium–glucose cotransporter-2 inhibitors.

**Table 2:** Changes in METS-IR and SPISE after 6 months of therapy and responder analyses according to treatment group.

Variable	GLP-1RA (Baseline)	GLP-1RA (6 Months)	Δ	SGLT2i (Baseline)	SGLT2i (6 Months)	Δ	*p*-Value (Δ)
METS-IR	56.07 ± 13.41	52.14 ± 11.99	−3.93 ± 5.92	46.06 ± 9.13	43.58 ± 8.87	−2.48 ± 2.63	0.115
SPISE	4.21 ± 1.47	4.67 ± 1.70	+0.46 ± 0.52	5.29 ± 1.41	5.73 ± 1.74	+0.44 ± 0.61	0.870
**Variable**	**GLP-1RA (*n* = 54)**			**SGLT2i (*n* = 46)**		***p*-value**	
METS-IR improvement, *n* (%)	44 (81.5)			33 (71.7)		0.257	
SPISE improvement, *n* (%)	45 (83.3)			33 (71.7)		0.167	
Improvement in both indices, *n* (%)	43 (79.6)			29 (63)		0.081	

Values are presented as mean ± Standard Deviation. Δ represents change from baseline to 6 months. *p*-values represent between-group comparisons of Δ values. Improvement was defined as a decrease in METS-IR and an increase in SPISE from baseline to 6 months, respectively. *p*-values for responder analyses were derived from χ^2^ tests. Abbreviations: Δ: change; METS-IR: Metabolic Score for Insulin Resistance; SPISE: Single Point Insulin Sensitivity Estimator; GLP-1RA: glucagon-like peptide-1 receptor agonist; SGLT2i: sodium–glucose cotransporter-2 inhibitor.

**Table 3:** Stratified and correlation analyses.

Analysis	GLP-1RA	SGLT2i	*p*-Value (Between Groups)
ΔMETS-IR (BMI ≥ 30 kg/m^2^)	−4.9 ± 6.1	−2.7 ± 2.9	0.089
ΔMETS-IR (BMI < 30 kg/m^2^)	−2.1 ± 4.0	−2.3 ± 2.4	0.812
ΔSPISE (BMI ≥ 30 kg/m^2^)	+0.52 ± 0.56	+0.47 ± 0.60	0.742
ΔSPISE (BMI < 30 kg/m^2^)	+0.38 ± 0.47	+0.42 ± 0.59	0.801
ΔMETS-IR vs. ΔBMI	0.48, *p* < 0.001	0.21, *p* = 0.180	0.041
ΔMETS-IR vs. ΔHbA1c	0.29, *p* = 0.030	0.12, *p* = 0.410	0.278
ΔSPISE vs. ΔBMI	−0.05, *p* = 0.72	−0.05, *p* = 0.75	0.980
ΔSPISE vs. ΔHbA1c	0.02, *p* = 0.91	−0.09, *p* = 0.60	0.760

Abbreviations: Δ: change from baseline to 6 months; BMI: body mass index; METS-IR: Metabolic Score for Insulin Resistance; SPISE: Single Point Insulin Sensitivity Estimator; HbA1c: glycated haemoglobin; GLP-1RA: glucagon-like peptide-1 receptor agonist; SGLT2i: sodium–glucose cotransporter-2 inhibitor.

## Data Availability

The data presented in the study are available on request from the corresponding author. The data are not publicly available due to privacy restrictions of the Greek National Health System.

## References

[1] Siam N.H., Snigdha N.N., Tabasumma N., Parvin I. (2024). Diabetes Mellitus and Cardiovascular Disease: Exploring Epidemiology, Pathophysiology, and Treatment Strategies. Rev. Cardiovasc. Med..

[2] Hussain A. (2025). Chronic hyperglycemia and cardiovascular dysfunction: An in-depth exploration of metabolic and cellular pathways in type 2 diabetes mellitus. Cardiovasc. Diabetol. Endocrinol. Rep..

[3] Soheilifard S., Faramarzi E., Mahdavi R. (2025). Association between cardiometabolic phenotypes with atherogenic index of plasma: A cross-sectional study from the Azar cohort population. BMC Cardiovasc. Disord..

[4] Marx N., Federici M., Schütt K., Müller-Wieland D., Ajjan R.A., Antunes M.J., Christodorescu R.M., Crawford C., Di Angelantonio E., Eliasson B. (2023). 2023 ESC Guidelines for the management of cardiovascular disease in patients with diabetes. Eur. Heart J..

[5] Koufakis T., Liberopoulos E.N., Kotsa K. (2022). A Horse, a Jockey, and a Therapeutic Dilemma: Choosing the Best Option for a Patient with Diabetes and Coronary Artery Disease. Am. J. Cardiovasc. Drugs.

[6] Tam C.S., Xie W., Johnson W.D., Cefalu W.T., Redman L.M., Ravussin E. (2012). Defining insulin resistance from hyperinsulinemic-euglycemic clamps. Diabetes Care.

[7] Bilbie Lupchian L., Oliván-Blázquez B., Peña-Galo E., Domínguez-García M., Sánchez-Calavera M.A. (2026). Comparison of four diagnostic indices for metabolic syndrome in a Northern Spanish population. PLoS ONE.

[8] Song K., Lee E., Lee H.S., Lee H., Lee J.W., Chae H.W., Kwon Y.J. (2025). Comparison of SPISE and METS-IR and Other Markers to Predict Insulin Resistance and Elevated Liver Transaminases in Children and Adolescents. Diabetes Metab. J..

[9] García Samuelsson M., Tárraga López P.J., López-González Á.A., Paublini H., Martínez-Almoyna Rifá E., Ramírez-Manent J.I. (2025). Assessment of the Risk of Insulin Resistance in Workers Classified as Metabolically Healthy Obese. Nutrients.

[10] Barchetta I., Dule S., Bertoccini L., Cimini F.A., Sentinelli F., Bailetti D., Marini G., Barbonetti A., Loche S., Cossu E. (2022). The single-point insulin sensitivity estimator (SPISE) index is a strong predictor of abnormal glucose metabolism in overweight/obese children: A long-term follow-up study. J. Endocrinol. Investig..

[11] Voziki D., Dimakopoulos G., Stergiou I., Zografou I., Rizzo M., Liberopoulos E.N., Kotsa K., Koufakis T. (2025). The effects of SGLT2 inhibitors and GLP-1 receptor agonists on the triglyceride to HDL cholesterol ratio and the triglyceride-glucose index in patients with type 2 diabetes. J. Diabetes Complicat..

[12] American Diabetes Association Professional Practice Committee for Diabetes* (2026). 2. Diagnosis and Classification of Diabetes: Standards of Care in Diabetes-2026. Diabetes Care.

[13] Bello-Chavolla O.Y., Almeda-Valdes P., Gomez-Velasco D., Viveros-Ruiz T., Cruz-Bautista I., Romo-Romo A., Sánchez-Lázaro D., Meza-Oviedo D., Vargas-Vázquez A., Campos O.A. (2018). METS-IR, a novel score to evaluate insulin sensitivity, is predictive of visceral adiposity and incident type 2 diabetes. Eur. J. Endocrinol..

[14] Paulmichl K., Hatunic M., Højlund K., Jotic A., Krebs M., Mitrakou A., Porcellati F., Tura A., Bergsten P., Forslund A. (2016). Modification and Validation of the Triglyceride-to-HDL Cholesterol Ratio as a Surrogate of Insulin Sensitivity in White Juveniles and Adults without Diabetes Mellitus: The Single Point Insulin Sensitivity Estimator (SPISE). Clin. Chem..

[15] Rizzo M., Rizvi A.A., Patti A.M., Nikolic D., Giglio R.V., Castellino G., Li Volti G., Caprio M., Montalto G., Provenzano V. (2016). Liraglutide improves metabolic parameters and carotid intima-media thickness in diabetic patients with the metabolic syndrome: An 18-month prospective study. Cardiovasc. Diabetol..

[16] Heinsberg L.W., Weeks D.E. (2022). Post hoc power is not informative. Genet. Epidemiol..

[17] Fazio S., Mercurio V., Tibullo L., Fazio V., Affuso F. (2024). Insulin resistance/hyperinsulinemia: An important cardiovascular risk factor that has long been underestimated. Front. Cardiovasc. Med..

[18] Janus A., Szahidewicz-Krupska E., Mazur G., Doroszko A. (2016). Insulin Resistance and Endothelial Dysfunction Constitute a Common Therapeutic Target in Cardiometabolic Disorders. Mediat. Inflamm..

[19] Mancusi C., Izzo R., di Gioia G., Losi M.A., Barbato E., Morisco C. (2020). Insulin Resistance the Hinge Between Hypertension and Type 2 Diabetes. High Blood Press. Cardiovasc. Prev..

[20] Bołdys A., Bułdak Ł., Nicze M., Okopień B. (2025). Liraglutide Reduces Liver Steatosis and Improves Metabolic Indices in Obese Patients Without Diabetes: A 3-Month Prospective Study. Int. J. Mol. Sci..

[21] Iordan L., Lazar S., Timar R., Popescu S., Sorescu T., Albai O., Braha A., Timar B., Gaita L. (2025). The Impact of Sodium-Glucose Co-Transporter-2 Inhibition on Insulin Resistance and Inflammation in Patients with Type 2 Diabetes: A Retrospective Study. Medicina.

[22] Hoffmann K., Michalak M., Paczkowska A. (2025). Relative Effectiveness and Safety of the GLP-1 (Glucagon-Like Peptide 1) Receptor Agonists, Semaglutide and Liraglutide in the Treatment of Obese Type 2 Diabetics: A Prospective Observational Cohort Study in Poland. Diabetes Metab. Syndr. Obes..

[23] Seufert J. (2015). SGLT2 inhibitors—An insulin-independent therapeutic approach for treatment of type 2 diabetes: Focus on canagliflozin. Diabetes Metab. Syndr. Obes..

[24] Correa-Burrows P., Matamoros M., de Toro V., Zepeda D., Arriaza M., Burrows R. (2023). A Single-Point Insulin Sensitivity Estimator (SPISE) of 5.4 is a good predictor of both metabolic syndrome and insulin resistance in adolescents with obesity. Front. Endocrinol..

[25] Herder C., Rizzo M., Roden M. (2024). Precision diabetology: Where do we stand now?. J. Diabetes Complicat..

[26] Kawai T., Autieri M.V., Scalia R. (2021). Adipose tissue inflammation and metabolic dysfunction in obesity. Am. J. Physiol. Cell Physiol..

[27] Kumar V. (2025). GLP-1/GLP-1R axis: From metabolism (obesity and T2DM) to immunity. Open Biol..

[28] Kyriakidou A., Koufakis T., Gika H., Kotsa K. (2025). Metabolomics Insights into the Benefits of SGLT2 Inhibitors in Type 2 Diabetes. Clin. Pharmacol..

[29] Renna N.F., Ramirez E.I., Arrupe M.F., Ramirez J.M. (2026). Ambulatory blood pressure monitoring strengthens the cardiovascular signal of GLP-1RA: A meta-analysis of blood pressure and weight mediation. J. Hypertens..

[30] Wanner C., Nangaku M., Kraus B.J., Zinman B., Mattheus M., Hantel S., Schumacher M., Ohneberg K., Schmoor C., Inzucchi S.E. (2024). How do SGLT2 inhibitors protect the kidney? A mediation analysis of the EMPA-REG OUTCOME trial. Nephrol. Dial. Transplant..

